# HLA Class I and Class II Associations in Dengue Viral Infections in a Sri Lankan Population

**DOI:** 10.1371/journal.pone.0020581

**Published:** 2011-06-09

**Authors:** Gathsaurie Neelika Malavige, Tim Rostron, Lochana T. Rohanachandra, S. D. Jayaratne, Neluka Fernando, Aruna Dharshan De Silva, Malaka Liyanage, Graham Ogg

**Affiliations:** 1 Department of Microbiology, University of Sri Jayawardanapura, Nugegoda, Sri Lanka; 2 Department of Medicine, University of Sri Jayawardanapura, Nugegoda, Sri Lanka; 3 MRC Human Immunology Unit, Weatherall Institute of Molecular Medicine, University of Oxford, Oxford, United Kingdom; 4 Genetech Research Institute, Colombo, Sri Lanka; 5 Department of Dermatology, Churchill Hospital, Oxford, United Kingdom; University of Rochester, United States of America

## Abstract

**Background:**

HLA class I and class II alleles have been shown to be associated with the development of dengue hemorrhagic fever (DHF)/dengue shock syndrome (DSS) in different populations. However, the majority of studies have been based on limited numbers of patients. In this study we aimed to investigate the HLA-class I and class II alleles that are positively and negatively associated with the development of DSS in a cohort of patients with DHF and also the alleles associated with development of DHF during primary dengue infections in a Sri Lankan population.

**Methodology/Principal Findings:**

The allele frequencies of HLA class I and class II alleles were compared in 110 patients with DHF and 119 individuals from the population who had never reported a symptomatic dengue infection at the time of recruitment. We found that HLA-A*31 (corrected P = 0.01) and DRB1*08 (corrected P = 0.009) were associated with susceptibility to DSS when infected with the dengue virus, during secondary dengue infection. The frequency of DRB1*08 allele was 28.7 times higher than in the normal population in patients with DSS. HLA-A*31 allele was increased 16.6 fold in DHF who developed shock when compared to those who did not develop shock. A*24 (corrected P = 0.03) and DRB1*12 (corrected P = 0.041) were strongly associated with the development of DHF during primary dengue infection.

**Conclusions/Significance:**

These data suggest that certain HLA alleles confer susceptibility/protection to severe dengue infections. As T cell epitope recognition depend on the HLA type of an individual, it would be now important to investigate how epitope specific T cells associate with primary and secondary dengue infections and in severe dengue infections.

## Introduction

Dengue viral infections have become one of the most important mosquito borne viral infections in the world and is one of the major emerging infectious diseases. In the past fifty years, its incidence has increased 30-fold with significant outbreaks occurring in five of six WHO regions. It is estimated that 2.1 million cases of dengue haemorrhagic fever (DHF)/dengue shock syndrome (DSS) occur every year resulting in 21,000 deaths [1].

Dengue viral infections may be caused by any of the four dengue virus serotypes (DEN1–4) which are closely related [2]. Initial infection with a particular serotype is known as primary infection, which is usually asymptomatic or results in mild disease manifestations [3]. However, subsequent infection with other serotypes (secondary dengue infections) may lead to severe disease which manifests in the form of DHF/DSS [3]. Currently, the pathophysiology of dengue viral infections and factors that result in severe clinical disease is poorly understood. Cross reactive memory T cells and cross reactive antibodies have been suggested to contribute to immunopathology by altering the cytokine profiles during secondary infection and are also believed to be less effective in eliminating the newly infective virus serotype [4], [5]. Therefore, they are thought to lead to enhanced viral replication and thus severe clinical disease.

Although cross reactive T cells and cross reactive antibodies may contribute to disease pathogenesis, these mechanisms alone do not explain the immunopathological mechanisms leading to severe disease, as severe clinical disease is known to occur even during primary dengue viral infections, especially infants [6] and in pregnant women [7]. Furthermore, it is believed that only 0.18–1% of primary infections and 2–9% of secondary infections manifest as DHF/DSS [3], and that the majority of individuals who are infected with the dengue virus develop mild or asymptomatic disease. Therefore, other factors are likely to play a significant role in the disease pathogenesis. Several genetic factors have been shown to be associated with the development of DHF/DSS and some have been shown to be protective [8], [9], [10]. Certain HLA- class I and class II alleles [8], [9], [11], [12], polymorphisms in the tumor necrosis factor alpha (TNF-α), Vitamin D receptor [13], CTLA-4, and transforming growth factor ß (TGF-β) [14] have been shown to be associated with development of DHF/DSS [10]. Alleles such as HLA-A*24, HLA-B*53, have been shown to increase the risk of DSS whereas HLA-DRB1*09, HLA-DR4, HLA-A*03 and HLA-B*18 have been found to decrease the risk of developing DSS [8], [9], [15]. Certain HLA alleles such as HLA-A*51, HLA-A*207 have been shown to increase the risk of severe DHF only during secondary dengue (SD) infection, while certain alleles such as HLA-B44, B62, B76 and B77 appeared to offer some protection [12]. Although Lan *et a*l have also tried to identify HLA alleles, which may increase the susceptibility for the development of DSS/severe DHF during primary dengue (PD) infection, some of the patients who had a PD infection were below the age of 1 year. Therefore, the presence of maternal antibodies in these babies could have skewed some of the results and contributed to occurrence of DHF [8]. These studies are important but have been limited in most cases by relatively small patient groups.

In this study we aimed to identify possible HLA- class I and class II alleles, which increase the risk of developing DHF/DSS during PD and SD infection in a cohort of individuals with DHF. We found that HLA-A*24 and HLA-DRB1*12 were associated with a significantly higher risk of developing DHF during PD infection. In addition, we also found that HLA-A*31 and HLA-DRB1*08 were significantly associated with a higher risk of developing DSS. These data would support the hypothesis that T cells contribute to the development of severe clinical disease during dengue viral infections.

## Methods

### Study population

110 patients with clinical features suggestive of dengue infections were who were admitted to a general medical ward in a tertiary care hospital in Colombo were enrolled in the study following informed written consent. The study was approved by the Ethical Review Committee of the University of Sri Jayawardanapura and the Ethical review Committee of the University of Oxford. Serial recordings of their clinical features and laboratory investigations (platelet counts, haematocrits, white cell counts) were made until they were discharged from the hospital in order to determine the severity of dengue infection. Patients with mild dengue/dengue fever were excluded from the study, and only patients with dengue haemorrhagic fever (DHF) (those who had evidence of plasma leakage) were recruited. These patients were classified as having dengue with warning signs (moderately severe dengue) and severe dengue according to the 2009 WHO guidelines [16]. Patients with DHF with less than or equal to a pulse pressure of 20 mmHg were classified as having shock [16].

HLA types of the normal population was derived from a study previously done by us [17]. As only 102 individuals (who were recruited from the Colombo district) were included in our previous study, we recruited 17 more healthy individuals who so far never had a symptomatic/clinically diagnosed dengue infection from the Colombo district. Therefore, HLA types of 119 individuals from the population were available for comparison with the dengue patients.

### Serology

Dengue virus infection was confirmed by testing the serum samples which were collected after day 6 of illness with a commercial capture-IgM and IgG enzyme-linked immunosorbent assay (ELISA) (Panbio, Brisbane, Australia). The ELISA was performed and the results were interpreted according to the manufacturer's instructions. This ELISA assay has been validated as both sensitive and specific for primary and secondary dengue virus infections [18]. Patients who only had dengue virus specific IgM were classified as having a PD infection while those who had a positive result for both IgM and IgG were classified as having a SD infection [16].

### HLA typing

HLA class I and class II alleles were typed as previously described [19]. DNA was extracted from whole EDTA blood samples from the patients using the QIAamp DNA blood Mini Kit (QIAGEN, UK). Extracted DNA was amplified and DNA typing was undertaken using a 144 sequence-specific primer (SSP) reactions to simultaneously detect all known HLA-A, B, C, DRB1, DRB3, DRB4, DRB5 and DQB1 specificities in an allele specific or group specific manner using the same method, reagents, PCR parameters and protocols for all loci. The PCR products were electrophoresed in 1.0% agarose gels, using xylene cyanol FF and bromophenol blue as marker dyes. The gels were run for 15 minutes at 15 V/cm in the 0.5×TBE buffer and visualized using UV illumination [20]. A phototype was considered to be successful when the control amplifications are positive and at least one allele or group was present in each locus. Allele frequencies were estimated from the number of positive typing reactions divided by the total number of haplotypes tested.

### Statistical analysis

The frequencies of HLA class I alleles and HLA class II alleles in patients with DHF and the normal population were compared to determine possible association with DHF. Graphpad Prism version 4 was used for statistical analysis. For each HLA allele, degree of association between HLA alleles and disease state was expressed as the odds ratio (OR), which is obtained from standard contingency table analysis by Haldane's modification of Woolf's method. The Fisher's exact test was used to determine the p value. The P values were further corrected by using the Bonferroni's inequality method [21]. The corrected P value (Pc) was calculated by multiplying the p values with the number of alleles tested for each locus. Comparisons to determine possible alleles that confer susceptibility to primary dengue infections were done by comparing the allele frequencies of patients with DHF during primary infection (n = 18) and patients with DHF during secondary dengue infection (n = 92). Comparisons to determine possible alleles that confer susceptibility to occurrence of shock during DHF were done by comparing the allele frequencies of patients with DHF associated with shock (n = 29) and patients with DHF who did not progress to shock (n = 81). Only the corrected p values were used to determine if certain HLA alleles were either positively or negatively associated with primary dengue or DSS. All statistical analysis and calculation of P values were done as described above [9], [21].

## Results

### Clinical characteristics and severity of dengue in the study population

Of the 110 patients with laboratory confirmed dengue infection, 32 (29.1%) were females. All patients had laboratory evidence of a rising haematocrit (evidence of plasma leakage) with a concurrent drop in platelet counts. 50 (45.45%) complained of abdominal pain and 49 (44.5%) had at least one bleeding manifestation. Based on the 2009 WHO diagnostic criteria, shock was defined as lowering of pulse pressure to 20 mmHg or less. Accordingly, 29 patients were classified as having shock. Of the 29 patients who developed shock, 23 (79.3%) had at least one bleeding manifestation and 5 (17.2%), had episodes of loss of consciousness. 20 (68.9%), of those who developed shock had either haematemesis or melana or significant bleeding (vaginal bleeding, epistaxsis or prolonged bleeding). Their mean platelet counts were 24.2 (SD±16.7), whereas the mean platelet counts of those who did not develop shock was 35.78 (SD±35.82). Of the DHF patients who did not develop shock 26 (32.1%) had at least one bleeding manifestation and 6 (7.4%) complained of melana and 3 (3.7%) complained of vaginal bleeding. None of the DHF patients without shock had haematemesis or severe bleeding.

Based on the presence of dengue specific IgM but no IgG, 18 (16.4%) patients were found to have PD infection. The mean age of those with PD was 24.7 (SD±6.6) and those with SD was 30.1 (SD±11.4). 3 (16.6%) patients with PD and 26 (28.2%) of those with SD developed shock. Bleeding manifestations were present in 9 (50%) of those with PD and 40 (43.4%) of those with SD. However, only 3 (the 3 patients who developed shock) of those with PD had a significant bleeding manifestation (melana, haematemesis, prolong or large bleed) (Table 1).

**Table 1:** Clinical characteristics of DHF patients with primary and secondary dengue.

Clinical characteristic	Primary dengueN (%)	Secondary dengueN (%)	TotalN (%)
Abdominal pain	6 (33.3)	44 (47.8)	50 (45.5)
Bleeding manifestations	9 (50)	40 (43.5)	49 (44.5))
Petechiae	3 (16.7)	10 (25)	13 (11.8)
Gum bleeding	3 (16.7)	17 (18.5)	20 (18.2)
Prolonged bleeding from sites of venepuncture	2 (11.1)	16 (17.4)	18 (16.4)
Haematemesis	0 (0)	8 (8.6)	8 (7.3)
Melena	3 (16.7)	18 (19.6)	21 (19.1)
Vaginal bleeding	0 (0)	6 (6.5)	6 (5.4)
Pleural effusions	2 (11.1)	15 (16.3)	17 (15.4)
Ascites	1 (5.5)	15 (16.3)	16 (14.5)
Narrow pulse pressure (<20 mmHg)	3 (16.7)	26 (28.3)	29 (26.4)
Platelet count <20,000 cells/mm^3^	5 (27.8)	42 (45.6)	47 (42.7)

### Associations of HLA class I and II alleles with DHF

The HLA class I and II allele frequency in the normal population and patients with DHF is shown in table 2. Among the HLA-A alleles, although not statistically significant after correction for multiple comparisons, A*68 allele was associated with a reduced risk of developing DHF (Odds ratio 0.302, CI 0.1096 to 0.8341). HLA-Cw*03 allele was also associated with a reduced risk of developing DHF (odds ratio 0.544, CI 0.2876 to 1.028), which was again not statistically significant. HLA-DRB1*08 was strongly associated with a higher risk of developing DHF (odds ratio 10.11, CI 1.269 to 80.50). The frequency of this allele in the normal population was 0.42, whereas the frequency in patients with DHF was 4.09. However again the association was not statistically significant (Pc = 0.0935).

**Table 2:** Allele frequencies (AF) of the HLA types of normal population (who so far have not reported a symptomatic dengue infection) and patients with acute DHF.

HLA allele	Normal populationAF (%)	Patients with DHFAF (%)	Odds ratio	CI (95%)	P_C_ value
HLA*A					
01	10.08	13.18	1.345	0.7617 to 2.406	1.00
02	16.39	14.09	0.837	0.5016 to 1.397	1.00
03	3.78	5.00	1.322	0.5440 to 3.297	1.00
11	10.5	10.9	1.039	0.5766 to 1.888	1.00
24	19.75	24.55	1.322	0.8489 to 2.059	1.00
26	5.46	4.55	0.824	0.3538 to 1.920	1.00
30	1.68	2.73	0.757	0.2801 to 2.005	1.00
31	4.2	3.18	0.759	0.2801 to 2.005	1.00
33	19.33	18.64	0.956	0.5989 to 1.526	1.00
68	7.14	2.27	0.302	0.1096 to 0.8341	0.161
HLA*B					
07	7.56	8.18	1.082	0.5513 to 2.152	1.00
08	1.26	1.36	1.082	0.2162 to 5.425	1.00
13	2.1	4.09	1.988	0.6556 to 6.027	1.00
15	8.82	8.64	0.978	0.5101 to 1.871	1.00
18	1.26	1.82	1.451	0.3209 to 6.558	1.00
27	1.68	1.36	0.809	0.1789 to 3.656	1.00
35	10.92	15.45	1.490	0.8622 to 2.577	1.00
37	2.94	3.64	1.245	0.4439 to 3.494	1.00
38	1.68	0.91	0.537	0.09728 to 2.961	1.00
40	12.18	10.00	0.801	0.4450 to 1.441	1.00
44	10.92	11.82	1.093	0.6133 to 1.947	1.00
51	7.14	6.82	0.952	0.4630 to 1.954	1.00
52	5.04	5.91	1.183	0.5277 to 2.651	1.00
55	5.88	4.55	0.755	0.3281 to 1.736	1.00
57	9.24	8.64	0.928	0.4877 to 1.766	1.00
58	7.56	4.55	0.528	0.2626 to 1.290	1.00
HLA*Cw					
01	5.88	2.73	0.448	0.1692 to 1.189	1.00
03	12.61	7.27	0.544	0.2876 to 1.028	0.63
04	11.76	17.73	1.616	0.9562 to 2.731	0.852
06	14.29	15.0	1.059	0.6304 to 1.778	1.00
07	24.37	25.91	1.085	0.7112 to 1.656	1.00
08	3.36	3.18	0.945	0.3368 to 2.651	1.00
12	7.98	13.18	1.75	0.9506 to 3.222	1.00
14	2.94	1.36	0.456	0.1165 to 1.787	1.00
15	10.08	9.55	0.942	0.5078 to 1.744	1.00
16	2.94	2.73	0.925	0.3060 to 2.798	1.00
HLA-DRB1*					
01	5.88	5.00	0.842	0.3738 to 1.897	1.00
03	4.62	3.18	0.678	0.2581 to 1.782	1.00
04	6.72	8.64	1.312	0.6565 to 2.620	1.00
07	21.01	23.64	1.164	0.7491 to 1.808	1.00
08	0.42	4.09	**10.11**	1.269 to 80.50	**0.0935**
10	7.56	7.27	0.957	0.4760 to 1.930	1.00
11	2.10	4.09	1.988	0.6556 to 6.027	1.00
12	3.78	4.09	1.085	0.4227 to 2.786	1.00
13	13.87	8.64	0.587	0.3232 to 1.067	1.00
14	8.4	9.09	1.090	0.5696 to 2.086	1.00
15	21.43	21.36	0.991	0.6370 to 1.558	1.00
**HLA-DQB1**					
02	17.65	15.0	0.823	0.5004 to 1.355	1.00
03	20.59	28.64	1.548	1.008 to 2.378	0.20
05	28.15	27.27	0.957	1.008 to 2.378	1.00
06	29.41	26.82	0.879	0.5846 to 1.323	1.00

### Associations of HLA class I and II alleles with DHF during primary dengue infections

The HLA class I and II allele frequency in the normal population, patients with DHF with PD and SD is shown in table 3. The allele frequency of HLA-A*24 was 44.44 in DHF with PD infection, 20.65 in patients with DHF and SD infection and 19.75 in the normal population. This allele was associated with development of DHF during PD infection (odds ratio 3.074, CI 1.454 to 6.495) which was statistically significant (Pc = 0.03). Among the HLA-B alleles, although not statistically significant, HLA-B*15 and HLA-B*35 were also associated with a higher risk of developing DHF during PD infection (odds ratio 2.63 and 2.56 respectively). Interestingly, the HLA-B*51 allele was not present in the DHF with PD infection, whereas the allele frequency in DHF patients with SD infection was 8.15 and the frequency in the normal population was 7.14. HLA-Cw*04 allele was found at a higher frequency in DHF patients with PD (27.78) than in DHF patients with SD (15.76) and the normal population (11.76) and it was associated with a higher risk of developing DHF during PD (odds ratio 2.056, CI 0.8961 to 4.716). However, again this association was not statistically significant.

**Table 3:** Allele frequencies (AF) of the HLA alleles of normal population and patients with primary (PD) and secondary dengue (SD).

HLA allele	Normal populationAF (%)	Patients with PDAF (%)	Patients with SDAF (%)	Odds ratio	95% confidence intervals	Pc value
**HLA-A**						
01	10.08	11.11	13.59	0.7950	0.2589 to 2.441	1.00
02	16.39	8.33	15.22	0.5065	0.1453 to 1.766	1.00
11	10.5	5.56	11.96	0.4332	0.09720 to 1.930	1.00
24	19.7	44.44	20.65	**3.074**	1.454 to 6.495	**0.03**
26	5.46	2.78	4.89	0.5556	0.06816 to 4.528	1.00
33	19.33	13.89	19.57	0.6631	0.2409 to 1.825	1.00
**HLA-B**						
07	7.56	2.78	9.24	0.2807	0.04129 to 2.189	1.00
15	8.82	16.67	7.07	2.63	0.9275 to 7.462	0.576
35	10.92	27.78	13.04	2.564	1.100 to 5.977	0.243
40	12.18	11.11	9.78	1.153	0.3658 to 3.633	1.00
44	10.92	16.67	10.87	1.640	0.6081 to 4.423	1.00
51	7.14	0	8.15	0.1498	0.00876 to 2.563	0.835
**HLA-Cw**						
03	12.61	11.11	6.52	1.792	0.5434 to 5.908	1.00
04	11.76	27.78	15.76	2.056	0.8961 to 4.716	0.482
06	14.29	11.11	15.76	0.6681	0.2196 to 2.033	1.00
07	24.37	25.00	26.09	0.9444	0.4146 to 2.151	1.00
12	7.98	13.89	13.04	1.075	0.3809 to 3.035	1.00
**HLA-DRB1**						
01	5.88	11.11	3.8	3.161	0.8742 to 11.43	0.511
03	4.62	5.56	2.72	2.106	0.3922 to 11.31	1.00
04	6.72	8.33	8.7	0.9545	0.2631 to 3.463	1.00
07	21.01	13.83	25.54	0.4701	0.1727 to 1.280	1.00
12	3.78	13.89	2.17	**7.258**	1.846 to 28.54	**0.041**
13	13.87	16.69	7.07	2.631	0.9275 to 7.462	1.00
15	21.43	19.44	21.74	0.8690	0.3544 to 2.131	1.00

Among the HLA class II alleles, HLA-DRB1*12 was found at a higher frequency in DHF patients with PD (13.89), when compared to those with SD (2.17) and normal population (3.78). This allele was found to be significantly associated with (Pc = 0.041) a higher risk of developing DHF during PD (odds ratio 7.258, CI 1.846 to 28.54). HLA-DRB1*01 was also found at a higher frequency among DHF patients with PD (11.11) when compared to DHF patients with SD (3.8) and the normal population (5.88). This allele too was found to be associated with a higher risk of developing DHF during PD (odds ratio 3.161, CI 0.8742 to 11.43), but this association was not statistically significant (Pc = 0.511). The frequency of DQB1*03 was lower in patients with PD (19.4%), when compared to those with SD (30.4%) and the normal population (20.6%). However, this association was not statistically significant.

### Associations of HLA class I and II alleles with development of severe dengue (dengue shock syndrome)

The HLA class I and II allele frequency in the normal population, DHF who did not develop shock and DHF with shock is shown in table 4. HLA-A*31 allele was found to be significantly (Pc = 0.01) associated with the occurrence of shock (odds ratio 18.58, CI 2.185 to 158.0). This allele was increased 16.6 fold in DHF who developed shock when compared to those who did not develop shock. The HLA-A*26 allele was not found in any of the patients who developed shock, whereas the frequency of this allele in the normal population was 5.46 and in the DHF patients who did not develop shock was 6.17. Therefore, HLA-A*26 appeared to decrease the risk of developing shock (odds ratio 0.124, CI 0.007154 to 2.154). However, this association was not statistically significant (Pc = 0.463). Among the other HLA class I alleles, HLA-B*15 and HLA-B*51 were increased 2.5 fold in patients who developed shock when compared to those who did not. Although both alleles were associated with a higher risk of developing shock (odds ratio 2.792, CI 1.073 to 7.267), this association was not statistically significant (Pc = 0.3132). B*07 was found at a very low frequency in patients with shock (1.72), when compared to DHF patients without shock (10.49) and the normal population (7.56). It was negatively associated with development of DSS (odds ratio 0.149, CI 0.01945 to 1.151), but this association was not statistically significant (Pc = 0.285). Among the HLA-Cw alleles, Cw*04 was found at a higher frequency among patients with shock (20.7%), when compared to those without shock (16.7%) and the normal population (11.8%). Cw*15 was found at a lower frequency in patients with shock (5.2%), when compared to those without shock (11.1%), suggesting that it may be protective. However, both associations with Cw*04 and Cw*15 were not statistically significant.

**Table 4:** Allele frequencies (AF) of the HLA alleles of the normal population, patients with DHF who did not develop shock and patients who developed shock.

HLA allele	Normal populationAF (%)	Patients with DHF but no shockAF (%)	Patients with shockAF (%)	Odds ratio	95% confidence intervals	Pc value
**HLA-A**						
01	10.08	12.35	15.52	1.304	0.5567 to 3.055	1.00
02	16.39	13.58	15.52	1.169	0.5040 to 2.711	1.00
11	10.50	10.49	12.07	1.171	0.4589 to 2.986	1.00
24	19.75	27.16	17.24	0.5587	0.2601 to 1.200	1.00
26	5.46	6.17	00	0.1241	0.007154 to 2.154	0.463
31	4.2	0.62	10.34	**18.58**	2.185 to 158.0	**0.01**
33	19.33	19.75	15.52	0.7462	0.3321 to 1.676	1.00
**HLA-B**						
07	7.56	10.49	1.72	0.149	0.01945 to 1.151	0.285
15	8.82	6.17	15.52	2.792	1.073 to 7.267	0.3132
35	10.92	15.43	15.52	1.007	0.4393 to 2.306	1.00
40	12.18	10.49	8.62	0.8047	0.8047	1.00
44	10.92	12.35	10.34	0.8192	0.3117 to 2.153	1.00
57	9.24	6.17	15.52	2.792	1.073 to 7.267	0.3132
**HLA-Cw**						
03	12.16	7.41	6.9	0.9259	0.2863 to 2.995	1.00
04	11.76	16.67	20.69	1.304	0.6112 to 2.784	1.00
06	14.29	13.58	18.97	1.489	0.6720 to 3.301	1.00
07	24.37	25.93	25.86	0.9967	0.5024 to 1.977	1.00
12	7.98	14.81	8.62	0.5425	0.1967 to 1.496	1.00
15	10.08	11.11	5.17	0.4364	0.1236 to 1.541	1.00
**HLA-DRB1**						
04	6.72	9.88	5.17	0.4977	0.1395 to 1.776	1.00
07	21.01	22.22	27.59	1.333	0.6722 to 2.645	1.00
08	0.42	1.23	12.07	10.98	2.210 to 54.56	**0.009**
10	7.56	8.02	5.17	0.6252	0.1715 to 2.278	1.00
13	13.87	8.64	8.62	0.9973	0.3426 to 2.903	1.00
14	8.4	9.88	6.9	0.6759	0.2163 to 2.112	1.00
15	21.43	22.84	17.24	0.7038	0.3246 to 1.526	1.00

HLA-DRB1*08 was strongly associated (Pc = 0.009) with the development of shock (odds ratio 10.98, CI 2.210 to 54.56). The frequency of this allele among patients with shock was 12.07, whereas the frequency among DHF without shock was 1.23 and the frequency in the normal population was 0.42. HLA-DRB1*04 appeared to be protective against shock (odds ratio 0.49) as it was found at a lower frequency in patients with shock (5.2%), when compared to those without shock (9.9%). However, this association was not statistically significant. There were no differences in the frequencies of the HLA-DQB1 alleles in patients with or without shock or the normal population.

## Discussion

In this study we have identified HLA class I and class II alleles that are associated with the development of DHF during PD infection and SD infection and also alleles that are strongly associated with the development of DSS. The allele frequencies of patients with DHF were compared with the normal population, which at the time of recruitment had never reported a symptomatic/clinically diagnosed dengue infection. However, given that 72% of children aged 12 years from the Colombo district in Sri Lanka are seropositive to dengue [22] it is likely that the majority of individuals who consisted of our normal population would have had at least one dengue virus infection.

When comparing allele frequencies of patients with DHF and the normal population, HLA-DRB1*08 allele was associated with the higher risk of developing DHF. The frequency of this allele in the normal population was 0.42, whereas the frequency in patients with DHF was 4.09. This allele was very strongly associated with the development of DSS (odds ratio 10.98, CI 2.210 to 54.56). The frequency of this allele among patients with shock was 12.07, which is 28.7 times higher than in the normal population. HLA-A*31 allele too was found to be significantly (Pc = 0.01) associated with the occurrence of shock (odds ratio 18.58, CI 2.185 to 158.0). This allele was increased 16.6 fold in DHF patients who developed shock when compared to those who did not develop shock. Sierra *et al* also found that HLA-A*31 was associated with DHF, but they have not analyzed its association with DSS/severe clinical disease [23]. However, both HLA-DRB1*08 and HLA-A*31 were only associated with DSS/DHF in patients with secondary dengue infection, which possibly suggests that T cell epitopes directed to this allele could be highly cross reactive. Mapping and phenotyping T cell epitopes specific for this allele could possibly reveal disease mechanisms that contribute to severe dengue infections. Although described by others [12], we did not find any association with HLA-B*51 or B*15 [23] and severe dengue infections. However, although not statistically significant, HLA-B*15 and B*57 were found at a higher frequency and were positively associated with susceptibility to DSS (odds ratio 2.79).

HLA-A*24 was found to be strongly associated (Pc = 0.03) with the development of DHF during PD infection (odds ratio 3.074). The allele frequency of HLA-A*24 was 44.44 in DHF with PD infection and 19.75 in the normal population. Several studies have shown that A*24 was associated with severe dengue [8], [9], [24]. Although not statistically significant, Lan *et al* also found that A*24 was associated with a higher risk of developing DSS/DHF with PD when compared to SD [8]. The other studies that reported that A*24 was associated with a higher risk of developing DHF/DSS had not reported the association of this allele in DHF/DSS patients with PD or SD. Although we found that A*24 was associated with DHF during PD, it was not associated with the development of DSS. In fact the allele frequency in patients with DSS was 17.24, whereas the frequency in DHF patients without shock was 27.16. Simmons et al et al also reported that HLA-A*24 T cell epitopes in the very highly conserved dengue virus NS3 protein have been identified [20] and are thought to be highly cross reactive [8]. The cross reactivity of this epitope is thought to contribute to disease pathogenesis [8]. However, the possible contribution of this allele in the development of DHF during PD infection cannot be explained by the cross reactivity between A*24 specific dengue virus T cell epitopes. Although several studies describe the associations of HLA-class I alleles with the development DHF/DSS, only a few have also described the associations with HLA-class II alleles. HLA-DRB1*12 allele was found to be associated (odds ratio 7.26) with the development of DHF during PD infections. This allele was 6.4 times higher in DHF patients with PD infection when compared to those with SD infection.

In the overall comparison of HLA allele frequencies, although not statistically significant, HLA-A*68 was found to be associated with a reduced risk of developing DHF (Odds ratio 0.302, CI 0.1096 to 0.8341). This allele was also not detected in DHF with PD and the allele frequency was 1.72 in patients with DSS (frequency in normal population was 7.14). Appannna *et al* also found that this allele was found at a lower frequency in patients of Indian ethnic origin with DHF [9]. Again although not statistically significant, HLA-A*26 and HLA-B*07 were found at a very low frequency in patients with shock when compared to those who did not develop shock and the normal population. However, Appanna et al found that in the Malay population in Malaysia, HLA-A*26 was associated with a higher risk of developing DHF [9]. Appana *et al* also described that B *13 was associated with reduced susceptibility to DSS [9]. This allele was not detected among our DSS cohort, which probably could imply that it does reduce susceptibility to DSS. However, as the overall frequency of this allele in the Sri Lankan population was low, it was not included in the analysis.

La Fleur *et al* showed that in the Mexican population, HLA-DR4 was protective against development of DHF [15]. We too found that this allele was present at a lower frequency among patients with DSS. However this was not statistically significant. DRB1*07 allele has also been shown to be associated with protection in the Cuban population [23]. We found that the allele was seen at a lower frequency in patients with DHF during PD infection and that it was negatively associated with development of DHF during PD (0.47). However, this association was not statistically significant. Although Nguyen *et al* found that HLA-DRB1*09 was negatively associated with DSS, this allele was not detected in the Sri Lankan population.

This study was carried out during the largest ever dengue epidemic in Sri Lanka, which occurred during the years 2009 and early 2010. Although in this study we have investigated the association of HLA alleles with primary and secondary dengue and also in the development of shock, it would have been useful to determine if infection with a particular dengue virus serotype also predisposes to shock or DHF during PD and its association with particular HLA alleles. However, as only adult patients were recruited in the study, they only presented to hospital with at least 4 to 5 days of fever and were therefore, only recruited to our study on day 5 of illness. Therefore, they were unsuitable for testing for the infecting dengue virus serotype.

In summary, in a large cohort of affected individuals, we have identified HLA class I and class II alleles that are associated with the development of DHF during PD infection and SD infection and also alleles that are strongly associated with the development of DSS. We found that HLA-A*31 and DRB1*08 were associated with susceptibility to DSS when infected with the dengue virus, during SD infection. A*24 and DRB1*12 were strongly associated with the development of DHF during PD infection. As T cell epitopes to some of these alleles have already been identified, it would be now important to investigate how epitope specific T cells could contribute to disease pathogenesis in primary and secondary dengue infections. Overall these data support a role for T cells in the pathogenesis of dengue associated disease.
